# Some Facts Concerning a "New Disease."

**Published:** 1920-02-21

**Authors:** 


### ENCEPHALITIS LETHARGICA.

### Some Facts Concerning a "New Disease."

So great has been the readiness with which popular fancy has elaborated both the novelty and the interest supposedly attaching to what thousands of the public have, after consulting their morning paper, anxiously referred to as the "sleeping sickness," that it is perhaps not out of place briefly to sketch the recent history of this malady and to outline the main facts in our knowledge concerning it. It is a frequent occurrence nowadays that the layman's attention is zealously drawn to the dangers likely to beset his bodily health, but, in fairness to the medical profession as a whole, it is probable that many of the "reported" comments on this disease had best been left unpublished if they could not be attuned to the actual facts of the situation.

### The First Recorded Cases.

As far as England is concerned, recognition of the first cases dates from attention drawn to them by Dr. Wilfred Harris, of London, and Dr. Hall, of Sheffield, in the *Lancet* during April 1918. It is now common knowledge that from the symptoms originally noted a confusion was made with the condition known as botulism, which is a disease essentially due to poisoning after the ingestion of food containing the *B. botulinus*. At the time the very natural tinned-food scare began, and, with the best of intentions, the Local Government Board issued a circular directing the notice of the profession to the disease, framing it, however, in suitably non-committal terms as to the exact nature and cause of the malady. A steady increase in the number of cases and opportunity to study them resulted before long in rendering the hypothesis of botulism utterly untenable, and in any case it was everywhere recorded that cases occurred where no tinned, preserved, or questionable foods had been eaten. It was, indeed, something of a local error, and is best completely forgotten, inasmuch that during the previous year Vienna had been similarly attacked, while in Paris, where Netter reported them in detail, a similar series occurred, so that, when the results of widespread observation had been summarised, there was little doubt left that the malady arose from either a toxic or an infective invasion of the grey matter of the brain, and possibly in some instances of the spinal cord also. The infecting agent still remains unidentified, though it is presumably of microbic origin, and possibly a "filter passer."

It is beyond the scope of this note to enter into the details of attempted isolation of a specific germ. In Vienna a "strepto-diplococcus" was said to have been consistently obtained. It was, indeed, stated that similar and fatal conditions had, with this organism, been produced in apes, but these results have not been generally confirmed, and we believe we are justified in saying that at the present moment, both here and in France, minute examination of *post-mortem* tissues has completely failed to demonstrate any recognisable causal germ. Bacteriological examination of the cerebro-spinal fluid has apparently always given a negative result, while the cytology has departed remarkably little from the normal. On the other hand, a fairly definite pathology has been established for the local changes in the brain, although from their distribution *post-mortem* it is still doubtful whether the condition could after death be diagnosed therefrom. Briefly, we simply know that there is congestion of the superficial vessels, darkening of the grey matter over a very limited area, and that the condition is essentially an encephalitis, and not a meningitis.

With regard to this localisation, we may summarise Dr. Kinnier Wilson's clear description of a somewhat difficult subject, which is published in full in the current number of the *Medical Annual.* He considers the commonest grouping as a "peri-aqueductal distribution." Thus the symptoms referrable to the intrinsic and extrinsic ocular muscles are almost constant—paralysis of accommodation, diplopia, ophthalmoplegia, and ptosis. These are frequently the earliest signs, and the last to disappear in recovery. Next in frequency comes the involvement of lower cranial nuclei, producing the expressionless and paralysed facial musculature, and a similar state in the palate, tongue, larynx, or pharynx. More rarely the limbs are involved, from implication of the cortico-spinal paths. Theoretically any area of grey matter may be affected, but unquestionably motor symptoms are more frequent than sensory, the latter being rare and, as it were, accidental. It is suggested that this special choice of motor nuclei indicates a specific action of the causal virus, whatever it may be.

### The Clinical Picture.

As might be expected from such a condition the effect on the patient's actions and appearance during the progress of the disease is very irregular. Although, as the name encephalitis lethargica suggests, a condition of indifference is the most typical, yet there are frequent periods of restlessness or even delirium. Delusions and hallucinations are not uncommon, but those will occur in any toxic state. Many other symptoms are undoubtedly the indirect result of a general psychical clouding and in our own experience typical meningeal symptoms, though sometimes present, are usually very mild or absent.

This rough sketch of the pathology may lead us on to the discussion of how and to what extent this may prove to be a "new disease." It is obvious from the usually insidious onset, the gradual specific involvement of a series of main nuclei all the while masked by a condition of general lethargy, possibly with febrile intervals, that these cases may be readily *confused* with influenza; tuberculous meningitis, uræmia, typhoid, etc. We use the italics in that the diseases encephalitis lethargica and epidemic

**Epidemic Encephalitis**—(*continued*).

influenza have been frequently coupled together and with poliomyelitis as infections with possibly a common or at least related infective origin. We would, however, point out that when symptomatology alone can be considered, with or without *post-mortem* pathological findings, it is more than easy to build up a series of morbid states into which the various groups of which these various forerunners of influenza may be placed. Possibly the conditions favourable to one virus are more or less favourable to the others and there may be much overlapping in the clinical pictures produced, but the little of the minute pathology that is really known at present does not to us bear out a common causal origin.

For example, as Dr. Kinnier Wilson points out, "several considerations point strongly in the direction of a distinct and new virus for this epidemic disease. Prominent among these are the insignificant and minor changes in the cerebro-spinal fluid. In a recent paper Dr. Vaidya amply corroborated this contention, concluding from the examination of a large number of cases under his observation at the London Hospital that in the fluid the deviation from the normal is very small. This is certainly contrary to the usual experience in cases of ordinary poliomyelitis." Many other differences present themselves both in making this comparison and in connection also with influenza, but for these we would refer the reader to the communication read by Dr. Edwin Bramwell before the Edinburgh Medico-Chirurgical Society on February 4, and to the extensive discussion following it.

If here we may dare to make our own summary, we feel that on all the evidence at present available we are in these cases dealing with a specific infection, possibly allied to, but not identical with, that of either poliomyelitis or influenza. It is a disease, new in the sense that its recognition as claiming a distinct pathology is of relatively recent origin, but not "new" in the sense that it is a brand new scourge arrived to afflict the inhabitants of these isles. Above all, and it is almost unnecessary here to mention the fallacy, it is not "sleeping sickness," a term which has for many years been devoted to trypanosomiasis, a chronic febrile disease of tropical countries due to a flagellate protozoon conveyed in the bite of insects and existing, readily observable, in the patient's blood-stream.

Attention is drawn to these cases for their novelty and interest rather than any sinister meaning which their occurrence may have. Also the condition of the tissues, their reaction to the virus, the whole type of infection is in direct contrast to those seen in acute fulminating influenza and its death-dealing complications. But a few months ago there seemed suddenly to be published so many valuable observations on filter-passing infections, and we appeared to be on the verge of more than one epoch-making discovery in connection with infections of which encephalitis lethargica is probably one. Suddenly has this advance stopped—or so one may be led to think, but the explanation is probably that early last spring we were provided in the medical journals with the accumulated experiences of four and a-half years. Certainly we learnt much during that time, but no dead end has now been reached. The future holds much promise in this direction, especially with regard to the "filter passers," and there is no need to create undue scares as to our helplessness against their attacks on the human race.

